# Structured H∞ Control for Spacecraft with Flexible Appendages

**DOI:** 10.3390/e23080930

**Published:** 2021-07-22

**Authors:** Yuntian Zhang, Aiping Pang, Hui Zhu, Huan Feng

**Affiliations:** 1 College of Electrical Engineering, Guizhou University, Guiyang 550025, China; ee.ytzhang18@gzu.edu.cn (Y.Z.); gs.huizhu19@gzu.edu.cn (H.Z.); gs.hfeng20@gzu.edu.cn (H.F.); 2 Guizhou Provincial Key Laboratory of Internet + Intelligent Manufacturing, Guiyang 550025, China

**Keywords:** structured control, flexible spacecraft, prevent oscillations

## Abstract

Spacecraft with large flexible appendages are characterized by multiple system modes. They suffer from inherent low-frequency disturbances in the operating environment that consequently result in considerable interference in the operational performance of the system. It is required that the control design ensures the system’s high pointing precision, and it is also necessary to suppress low-frequency resonant interference as well as take into account multiple performance criteria such as attitude stability and bandwidth constraints. Aiming at the comprehensive control problem of this kind of flexible spacecraft, we propose a control strategy using a structured H-infinity controller with low complexity that was designed to meet the multiple performance requirements, so as to reduce the project cost and implementation difficulty. According to the specific resonant mode of the system, the design strategy of adding an internal mode controller, a trap filter, and a series PID controller to the structured controller is proposed, so as to achieve the comprehensive control goals through cooperative control of multiple control modules. A spacecraft with flexible appendages (solar array) is presented as an illustrative example in which a weighted function was designed for each performance requirement of the system (namely robustness, stability, bandwidth limit, etc.), and a structured comprehensive performance matrix with multiple performance weights and decoupled outputs was constructed. A structured H-infinity controller meeting the comprehensive performance requirements is given, which provides a structured integrated control method with low complexity for large flexible systems that is convenient for engineering practice, and provides a theoretical basis and reference examples for structured H-infinity control. The simulation results show that the proposed controller gives better control performance compared with the traditional H-infinity one, and can successfully suppress the vibration of large flexible appendages at 0.12 Hz and 0.66 Hz.

## 1. Introduction

With the rapid development of the aerospace industry and of composite material technology, along with its broad application in aerospace, the structure of spacecrafts is becoming larger and more flexible, featuring multi-system modalities. The resonant mode of a flexible system of this type leads to tremendous changes in amplitude features. Meanwhile, the inherent low-frequency interference caused by the complex launch environment and the high-altitude environment during orbit operation, as well as the flexible mode of the system, greatly limit the choice of bandwidths, i.e., robust stability. The flexible modes and low-frequency disturbances inherent in the high-altitude environment impair the stability and performance of the spacecraft, which will cause performance degradation and failure to meet mission requirements, or result in unstable control or even failure of the spacecraft.

With the increasing diversification of spacecraft missions, the requirements for pointing accuracy of large spacecraft have become increasingly stringent, which makes control research more complicated and difficult to delve into. The difficulties of the control design of such large flexible systems are as follows: to suppress the external interference caused by the complex space environment and the inherent low-frequency resonance interference of flexible spacecraft; to meet “high-precision” performance requirements; and to ensure attitude stability and bandwidth amount (robustness requirement).

Previous studies show that in terms of a synthesis control over these spacecraft with multi-performance requirements, it is difficult to apply classic analysis methods to balance the requirements. Traditional control design schemes, in general, fail to simultaneously meet the requirements of pointing accuracy and robustness [1,2,3,4,5]. All of them [6,7,8] used some fuzzy/neural control scheme to deal with at least two of these undesirable aspects: presence of inertia uncertainties, misalignment, unknown or external disturbance, vibration, actuator saturation, and faults, to ensure spacecraft stability. H∞ control theory is a comprehensive control theory that can take multiple performance requirements into consideration in the design and is suitable for such comprehensive control problems with multiple performance requirements. Currently, robust adaptive control, robust H∞ control, and μ synthesis control are mainly adopted to realize vibration control during the stable operation of flexible spacecraft [9,10,11,12,13]. 

Despite its synthesis advantages, H∞ design has engineering application limitations [14] mainly due to its high-order and complicated controller. Apart from high costs, it is a tremendous challenge to decompose a high-order and complicated controller into multiple low-complexity control structures based on experience in engineering practices [15,16], thus leading to a low feasibility of the practical use of traditional H∞ controllers. A new H∞ control method combining the advantages of the traditional H∞ control [15,16,17] was proposed by Apkarian in recent years, which factors into system performances in all aspects and overcomes the infeasibility of engineering applications of traditional H∞ controllers due to non-transparency and high complexity. The new method has attracted much attention and been widely applied since it was proposed [18,19,20]. The logic of the new structured H∞ control method is as follows: the structure of a controller is first designed according to actual needs and control objectives; on this basis, appropriate weighting functions are selected as per the specific performance requirements of the control object in order to form an H∞ performance matrix with multi-dimensional performance output; and finally, the structured H∞ controller with optimal parameters can be obtained through parameter optimization of the controller with a fixed structure [21,22,23].

In view of problems in integrated control of spacecraft with large flexible solar panels, based on the structured H∞ control design strategy and the specific resonance mode of the system, this paper proposes incorporating into the controller structure an internal mode controller, a notch filter, and a serial PID controller, which can achieve integrated control through multi-control module collaboration. A control solution that satisfies the comprehensive performance requirements is provided, thus reducing the project cost and implementation difficulty. Apart from the introduction, this paper deals with system modeling in the second part, structured H∞ controller design in the third part, performance simulation and analysis in the fourth part, and conclusions in the last part.

## 2. System Model Case and Control Analysis

**Figure 1:**
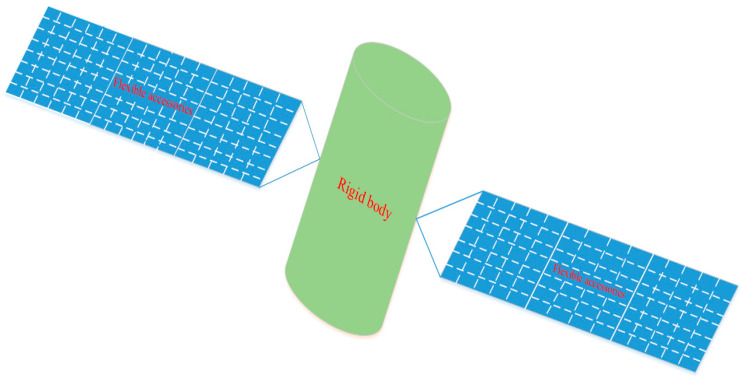
Spacecraft with large flexible appendages.

The spacecraft with large flexible appendages is shown in Figure 1. It mainly comprises solar arrays that provide energy, velocity gyroscopes, precision guidance sensors, and star trackers that provide spacecraft attitude data, as well as a reaction wheel and an electromagnetic torque device for momentum management, and a digital computer.

Only the pitch axis model that is most jitter-prone and the most important in the entire system is considered when constructing the simulation model. Official data show that the flexible spacecraft’s pitch axis model is composed of a rigid body model and several flexible modules, as shown in the following formula:

(1) $$\frac{\theta \left(s\right)}{u\left(s\right)}=\frac{1}{IS^{2}}+\sum_{i=1}\frac{K_{i}/I}{s^{2}+2\zeta w_{i}s+w_{i}^{2}}$$

In the formula, *θ* is the angular error of the pitch axis affected by the jitter of solar arrays; *u* is the given input of the pitch axis torque; *s* is the Laplace operator; *I* is the spacecraft pitch inertia constant with the value of 77,076 kg·$m^{2}$; *ξ* = 0.005 is the passive damping ratio constant of the system; *Ki* is the flexibility gain of the flexible module; and $\omega_{i}$ is the flexible frequency of the flexible module. The data are shown in Table 1.

**Figure 2:**
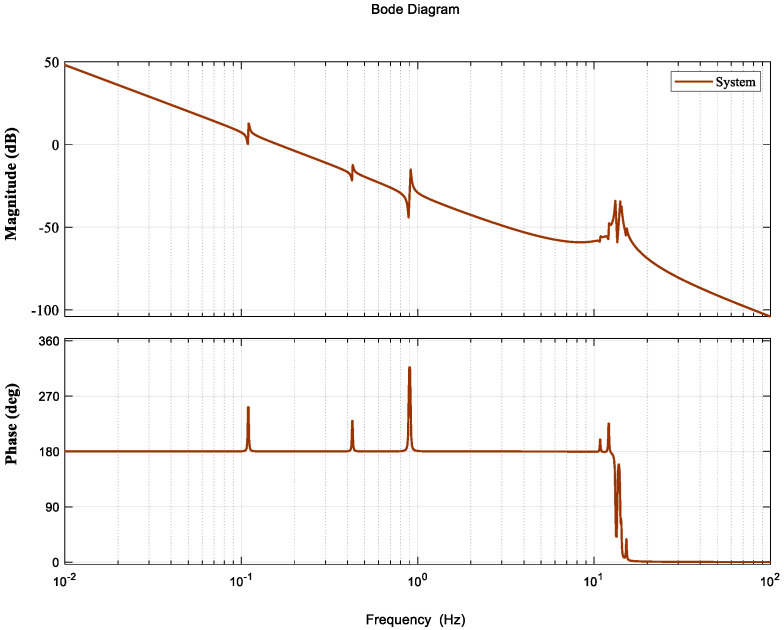
Bode plot without a control system.

The Bode plot of the system without a controller is shown in Figure 2. The Bode plot shows that the cut-off frequency $\omega_{c}$ is only 0.16 Hz and the bandwidths of the system are very small, indicating poor interference suppression.

Since they were put into operation, flexible spacecraft, as high-precision spacecraft, have never produced an output error of pitch axis exceeding 0.007 arcsec, which requires good control performance of the system. An effective controller should be good at disturbance suppression of solar panels and inherent flexibility suppression of the system, with a certain number of bandwidths.

The control redesign requirements can be stated as follows [24]:

1. Maintain at least 5 dB gain margin and 20 deg phase margin.
2. Provide at least 6 dB gain suppression (roll off) of the high-frequency spacecraft structural modes at 14 Hz.
3. Provide at least 20 dB additional disturbance attenuation at both 0.12 Hz and 0.66 Hz with respect to the original design.
4. Maintain the bandwidth (the open-loop gain crossover frequency) close to 1.5 Hz.

## 3. Design of Structured H∞ Control

In general, the complete design of structured control is divided into three steps: firstly, to design a structured controller according to design requirements and control objectives; secondly, to select and design proper weighting functions based on control objectives and performance requirements; and finally, to obtain a desired structured controller according to performance requirements and selected weighting functions. 

In Section 3.1, the author briefly analyzes the control objectives and performance requirements in the control design of the large flexible spacecraft in this case and presents controller structure design in terms of the flexible modes and disturbance model of the system.

### 3.1. Structured Controller Setting

The main problems confronted in the control of large flexible spacecraft are as follows: first, the multiple low-frequency resonance modes of the system result in huge changes in amplitude characteristics; second, the inherent low-frequency interference caused by the complex launch environment and the high-altitude environment during orbit operation, as well as the flexible mode of the system, greatly limit the choice of bandwidths. The controller is designed to suppress the inherent low-frequency resonance disturbance of the system and achieve high pointing accuracy, while ensuring the bandwidth amount of the flexible system (robustness) and stability.

**Figure 3:**
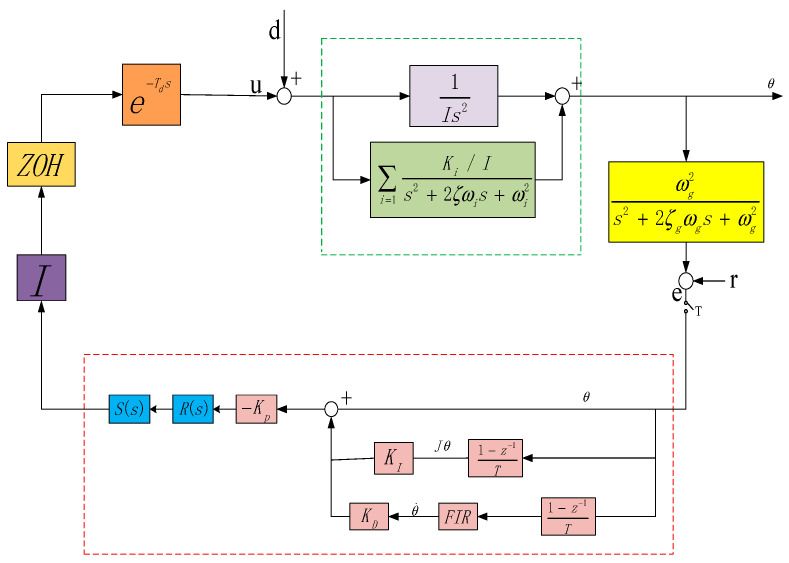
Control system block diagram.

To meet the above control target, based on the flexible mode and inherent disturbance frequency of the system in this case, the authors design a structured integrated controller as shown in Figure 3.

The dotted line in Figure 3 shows the structured controller of the system. The red modules are for adjustable parameters, and the blue modules are fixed parameters.

The structured H∞ controller consists of three parts. The first part is an internal mode controller *S*(*s*) designed for the system’s resonance modes to suppress resonance disturbance.

(2) $$S(s)=\frac{\left[{\left(\frac{s}{87}\right)}^{2}+\frac{0.002s}{87}+1\right]}{\left[{\left(\frac{s}{45}\right)}^{2}+\frac{1.4s}{45}+1\right]}$$

**Figure 4:**
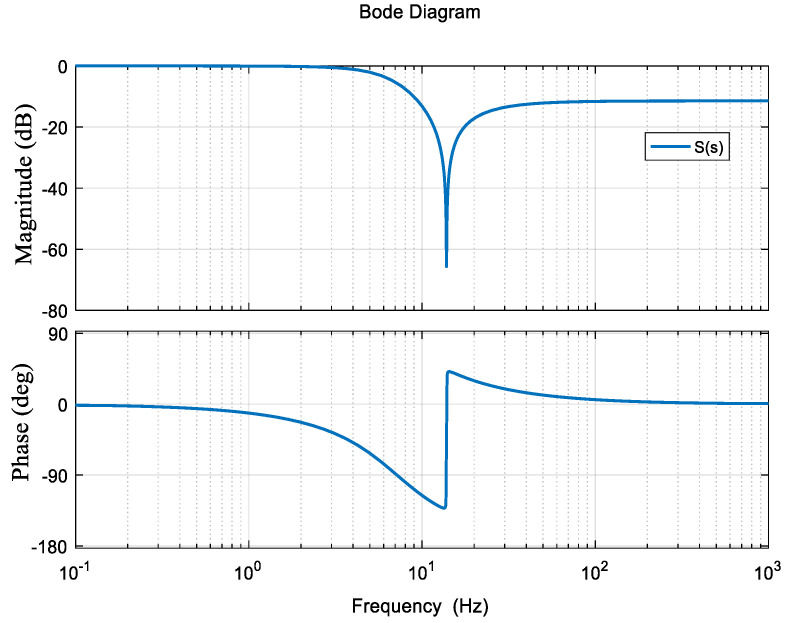
Internal mode controller *S*(*s*).

Its frequency characteristics are shown in Figure 4 and its purpose is to reduce system vibration by increasing the damping of the system for system vibration at 14 Hz.

**Figure 5:**
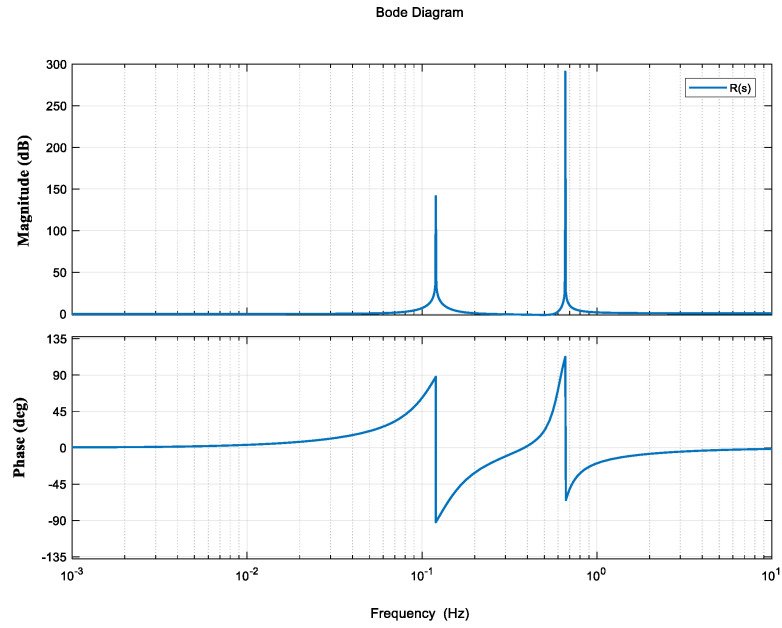
Notch filter *R*(*s*).

The second part is a notch filter *R*(*s*) set by the disturbance characteristics of the space environment to ensure sufficient suppression of the disturbance of solar panels at 0.12 Hz and 0.66 Hz on the premise of not affecting the stability of the frequency band in the system. The Bode plot is shown in Figure 5.

(3) $$R(s)=\frac{\left[{\left(\frac{s}{0.77872}\right)}^{2}+\frac{0.728s}{0.77872}+1\right]}{\left[{\left(\frac{s}{0.7536}\right)}^{2}+1\right]}\times \frac{\left[{\left(\frac{s}{3.84336}\right)}^{2}+\frac{0.254s}{3.84336}+1\right]}{\left[{\left(\frac{s}{4.1448}\right)}^{2}+1\right]}$$

The third part is an adjustable parameter PID controller set to ensure the stability of the system, whose rate path is supplemented with an FIR filter to provide gain suppression.

### 3.2. Selection of Performance Weighting Functions

The following indicators need to be factored into the selection of weighting functions: the requirement of pointing accuracy; the stability and sensitivity of the system after the internal model controller of disturbance suppression is added; and the bandwidth limitations (robustness) of the flexible system.

By setting $T_{1}$ as the transfer function of r−e, the stability of the system is the distance from the transfer function $T_{1}$ to the critical operating point, which is also the upper limit of the gain of tracking performance, requiring:

(4) $${\left\|W_{1}\left(s\right)T_{1}\left(s\right)\right\|}_{\infty }\leq \gamma$$

where γ is the norm index, and $W_{1}\left(s\right)$ is the weighting function. The upper limit of the design stability margin is 1, $W_{1}\left(s\right)=1$.

$T_{2}$ is set as the transfer function from *d* to *θ*, which is the robust stability requirement of the system:

(5) $${\left\|W_{2}\left(s\right)T_{2}\left(s\right)\right\|}_{\infty }\leq \gamma$$

where the weighting function $W_{2}\left(s\right)=0.8$.

By setting $T_{3}$ as the transfer function from *r* to *θ*, the bandwidth requirement of the system is:

(6) $${\left\|W_{3}\left(s\right)T_{3}\left(s\right)\right\|}_{\infty }\leq \gamma$$

**Figure 6:**
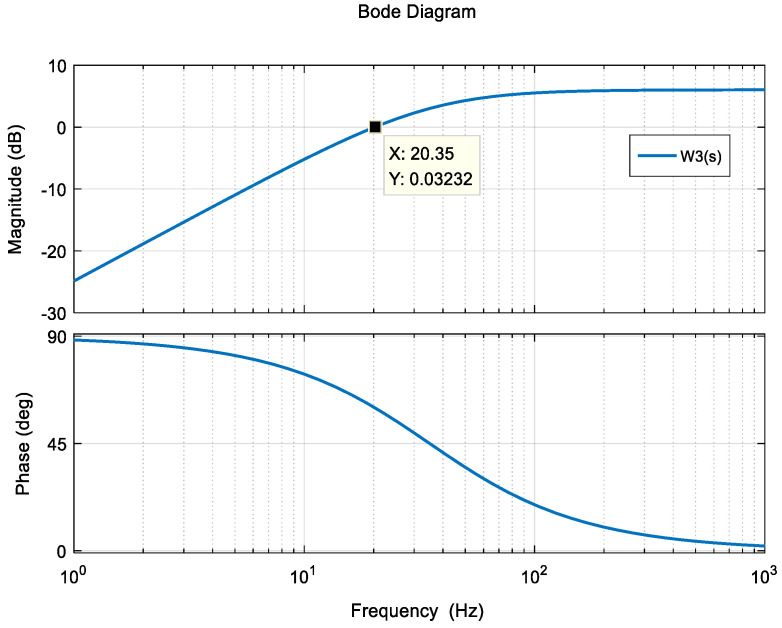
Bode plot of $W_{3}\left(s\right)$.

In order to limit the bandwidth of the system, the weighting function $W_{3}\left(s\right)$ can be selected in the high-pass filter form as follows: The Bode diagram for $W_{3}\left(s\right)$ is shown in Figure 6.

(7) $$\begin{array}{l} W_{3}\left(s\right)=\frac{2s}{s+220} \end{array}$$

### 3.3. Parameter Optimization of Structured Controller

Based on comprehensive considerations of the performance requirements of the system and the implementation cost of the controller, the controller was designed as shown in Formula (8).

(8) $$K(s)=K_{P}(1+K_{I}/s+K_{D}s)\times R(s)\times S(s)$$

$K_{P},K_{I},K_{D}$ is the to-be-optimized parameter.

Given the above analysis, and for the structured $H_{\infty }$ optimization of the designed controller structure and the weighting function, the control performance requirements of the flexible system was considered comprehensively, and the minimum $K_{P},K_{I},K_{D}$, and the minimum value satisfying Formula (9) can be obtained by optimizing the adjustable parameters.

(9) $${\left\|H\right\|}_{\infty }\leq \gamma$$

In formula $H=diag\left(\begin{array}{ccc} W_{1}T_{1} & W_{2}T_{2} & W_{3}T_{3} \end{array}\right)$, the adjustable parameter yielded is the optimal one for the system controller.

When seeking the optimal parameter of the structured controller $H_{\infty }$, the linear fractional transformation (LFT) [18,19,20,21,22,23,24] was employed with $T_{1}$, $T_{2}$, and $T_{3}$ in Formulas (4)–(6), and the structured controller *C* with parameters was extracted and expressed in the following linear fraction forms to optimize the parameters:

(10) $$\{\begin{array}{c} T_{1}{=F}_{l}\left(\begin{array}{cc} P_{1} & C \end{array}\right)\\ T_{2}{=F}_{l}\left(\begin{array}{cc} P_{2} & C \end{array}\right)\\ T_{3}{=F}_{l}\left(\begin{array}{cc} P_{3} & C \end{array}\right) \end{array}$$

In this case, the optimal parameters of the structured controller in Figure 1 yielded through repeated iterative calculations are as follows:

$$K_{P}=8,K_{I}=0.5,K_{D}=0.95$$

The final controller is:

(11) K(s)=8(1+0.5/s+0.95s)×R(s)×S(s)

## 4. Simulation Performance Analysis

**Figure 7:**
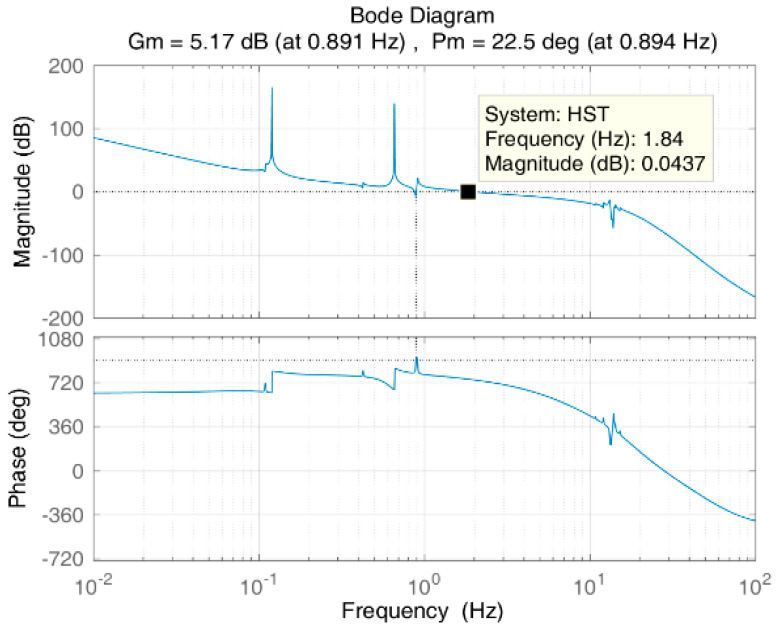
Open-loop Bode plot of the system.

Figure 7 shows the Bode plot of the unified open-loop frequency domain. According to Figure 7, the gain margin of the system is 5.17 dB; the phase margin is 22.5°; and the cut-off frequency is approximately 1.8 Hz, exceeding the required cut-off frequency of 1.5 Hz, which indicates that the stability and bandwidth requirements of the system have been met. For the system flexibility at 13 to 14 Hz, the controller provides about −15 to −55 dB gain suppression, which basically meets the suppression requirements for the inherent flexibility of the system.

**Figure 8:**
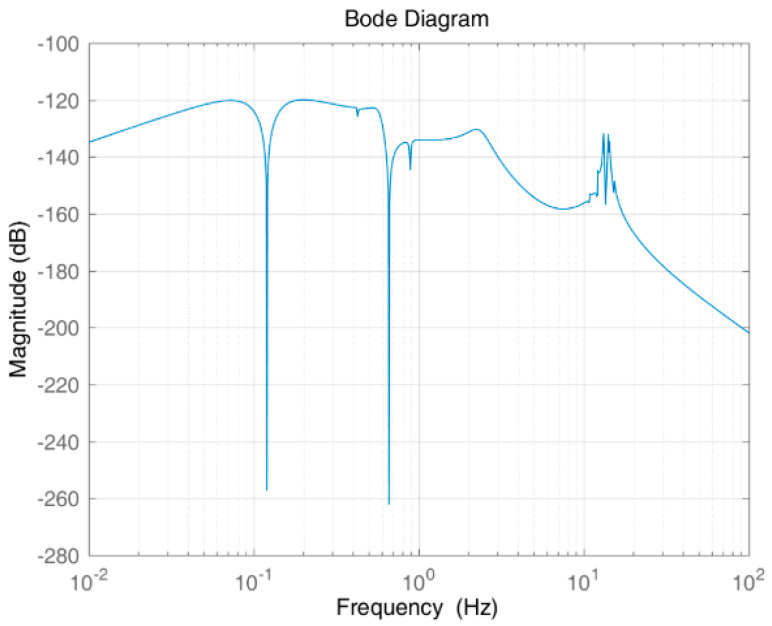
Closed-loop Bode plot of the system.

**Figure 9:**
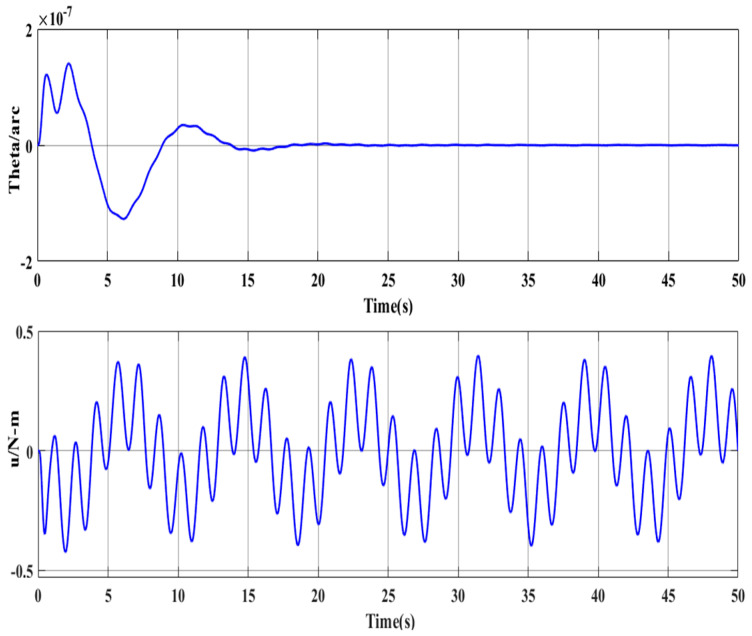
System time-domain response.

Figure 8 is a closed-loop Bode plot of the system. Figure 9 is the time-domain response of the system. The figure shows that the proposed controller provides gain attenuation far higher than 20 dB for the jitter at 0.12 Hz or 0.66 Hz, which is also proved by the time-domain response of the system shown in Figure 7.

With the same weight function, the obtained H-inf controller is as follows:

(12) $$K_{h}(s)=\frac{3.492\times 10^{7}\times (s+0.6402)(s^{2}+0.002009s+1.609\times 10^{-6})(s^{2}+0.0003605s+0.002128)}{{\left(s+0.01\right)}^{2}(s^{2}+0.00022s+0.002118)(s^{2}+3357s+3.763\times 10^{-6})}$$

**Figure 10:**
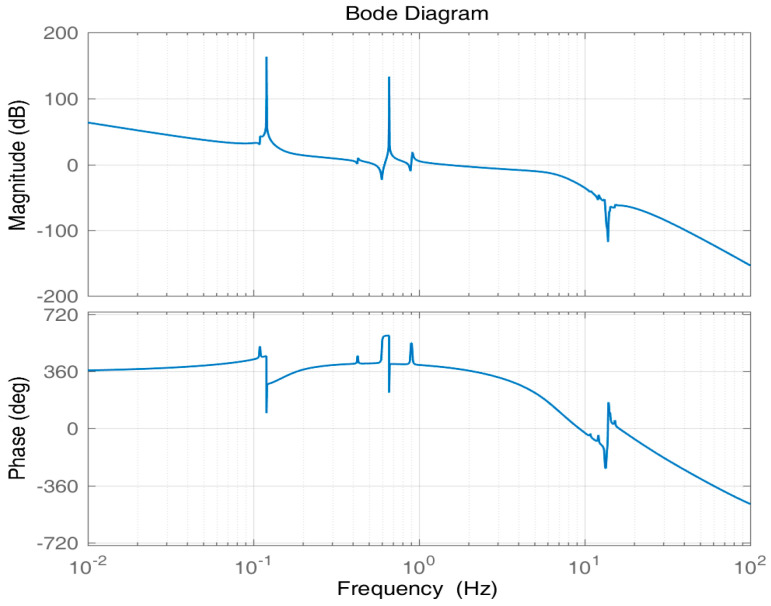
Open-loop Bode plot of H-inf controller.

The open-loop Bode plot of the H-inf controller is shown in Figure 10.

**Figure 11:**
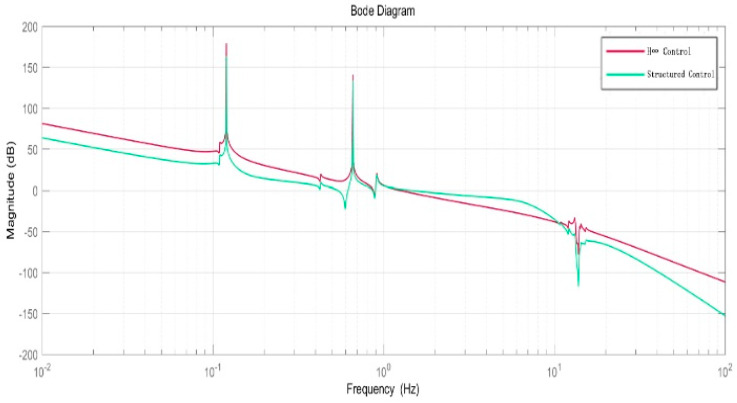
Open-loop simulation comparison between the structured integrated controller and traditional H-inf controller.

**Figure 12:**
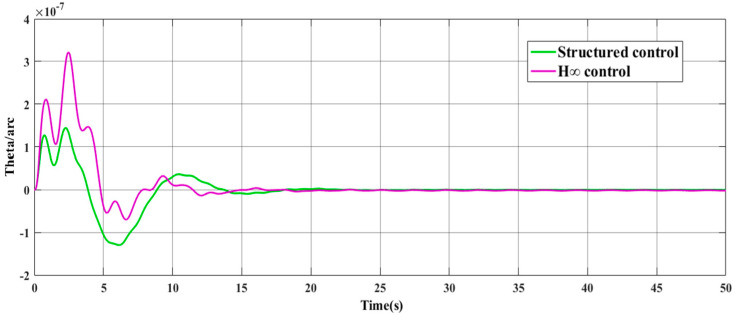
Simulation comparison of time-domain response between the structured integrated controller and traditional H-inf controller.

The frequency domain response shown in Figure 11 shows that the structured integrated controller is excellent in vibration suppression for the flexible structure at 0.12 Hz and 0.66 Hz. The time-domain response shown in Figure 12 shows that under the same weight function, both controllers (structured integrated controller and H-inf controller) can completely suppress solar panel disturbance within a certain period, meet the control requirements, and present stability. For the structured integrated controller, the overshoot of the system is 2.101%; the peak time is 0.711 s; and the adjustment time is 13.562 s. For the traditional H-inf controller, the overshoot is 2.31%; the peak time is 0.876 s; and the adjustment time is 14.227 s. The data clearly show that the structured integrated controller has significantly better dynamic performance than the H-inf controller and the structured integrated controller has a lower order.

## 5. Conclusions

To solve the comprehensive control problems of spacecraft with large flexible appendages such as insufficient bandwidths, low system directivity accuracy, and flexible structure vibration, this paper proposes a structured integrated controller that satisfies control requirements by selecting appropriate weight functions. The simulation results indicate that the proposed controller can effectively suppress the vibration of large flexible appendages at 0.12 Hz and 0.66 Hz. While ensuring high pointing accuracy, the structured integrated controller can meet the requirements of attitude stability and bandwidths. Compared with the traditional H-inf controller, the proposed controller has the advantages of lower complexity and system order as well as lower engineering costs and implementation difficulty, with better control performance.

## Figures and Tables

**Table 1:** Flexible spacecraft system model parameters.

$K_{i}/\mathrm{kg}\times m^{2}$	$\omega_{i}/\mathrm{Hz}$
0.018	0.110
0.012	0.432
0.057	0.912
0.024	10.834
0.155	12.133
−1.341	13.201
−1.387	14.068
−0.806	14.285
−0.134	15.264
